# The effects of pemafibrate and omega-3 fatty acid ethyl on apoB-48 in dyslipidemic patients treated with statin: A prospective, multicenter, open-label, randomized, parallel group trial in Japan (PROUD48 study)

**DOI:** 10.3389/fcvm.2023.1094100

**Published:** 2023-01-25

**Authors:** Yasutaka Takeda, Ichiro Sakuma, Shinya Hiramitsu, Mizuho Okada, Shinichiro Ueda, Masaru Sakurai

**Affiliations:** ^1^Division of Metabolism and Biosystemic Science, Gastroenterology, and Hematology/Oncology, Department of Medicine, Asahikawa Medical University, Asahikawa, Japan; ^2^Caress Sapporo Hokko Memorial Clinic, Sapporo, Japan; ^3^Hiramitsu Heart Clinic, Nagoya, Japan; ^4^Keiyukai Yoshida Hospital, Asahikawa, Japan; ^5^Department of Clinical Pharmacology and Therapeutics, University of the Ryukyus, Nishihara, Japan; ^6^Department of Social and Environmental Medicine, Kanazawa Medical University, Uchinada, Japan

**Keywords:** apolipoprotein B-48, atherosclerotic cardiovascular disease, residual risk, hypertriglyceridemia, pemafibrate, polyunsaturated fatty acids (PUFAs)

## Abstract

**Background:**

We compared the lowering effects of pemafibrate and omega-3 fatty acid ethyl on fasting apolipoprotein (apo) B-48 (apoB-48), a marker that reflects postprandial hypertriglyceridemia, which is one of the residual risks for atherosclerotic cardiovascular disease (ASCVD) with statin treatment.

**Methods:**

This prospective, multicenter, open-label, randomized, parallel group trial was conducted at 4 medical institutions between April 2020 and May 2022. A total of 126 ambulatory patients with dyslipidemia receiving statin treatment for more than 4 weeks, aged 20–79 years with fasting triglyceride (TG) levels of ≥177 mg/dl were randomly assigned to 16-week pemafibrate 0.4 mg per day treatment group (PEMA, *n* = 63) or omega-3 fatty acid ethyl 4 g per day treatment group (OMEGA-3, *n* = 63). The primary endpoint was the percentage change in fasting apoB-48 from baseline to week 16.

**Results:**

The percentage changes in fasting apoB-48 in PEMA and OMEGA-3 were −50.8% (interquartile range −62.9 to −30.3%) and −17.5% (−38.3 to 15.3%) (*P* < 0.001), respectively. As the secondary endpoints, the changes in fasting apoB-48 in PEMA and OMEGA-3 were −3.10 μg/ml (−5.63 to −1.87) and −0.90 μg/ml (−2.95 to 0.65) (*P* < 0.001), respectively. Greater decreases with significant differences in the percentage changes in TG, remnant lipoprotein cholesterol, apoC-III, fasting plasma glucose, alanine aminotransferase, gamma-glutamyl transpeptidase, and alkaline phosphatase were observed in PEMA, compared with OMEGA-3. Greater increases with significant differences in those in high-density lipoprotein (HDL) cholesterol, apoA-I, and apoA-II were observed in PEMA, compared with OMEGA-3. PEMA showed anti-atherosclerotic lipoprotein profiles in gel-permeation high-performance liquid chromatography analyses, compared with OMEGA-3. Although adverse events occurred in 9 of 63 (14.3%) patients in PEMA and 3 of 63 (4.8%) patients in OMEGA-3, no serious adverse events associated with drug were observed in either group.

**Conclusions:**

This is the first randomized trial to compare the lowering effects of pemafibrate and omega-3 fatty acid ethyl on fasting apoB-48. We concluded that pemafibrate was superior to omega-3 fatty acid ethyl in lowering effect of fasting apoB-48. Pemafibrate is expected to reduce the residual risk for ASCVD with statin treatment.

**Clinical trial registration:**

https://rctportal.niph.go.jp/en, identifier jRCTs071200011.

## 1. Introduction

Meta-analyses of large-scale clinical trials showed that low-density lipoprotein cholesterol (LDL-C)-lowering therapy with statin reduced the incidence of major cardiovascular events in proportion to the degree of LDL-C reduction, regardless of absolute risks in individuals such as sex differences, history of coronary artery disease (CAD), and LDL-C levels (1–4). Thus, there is no doubt that LDL-C lowering therapy is of great importance in the prevention of atherosclerotic cardiovascular disease (ASCVD). Although a 1 mmol/L reduction in LDL-C with statin treatment has been shown to reduce the incidence of major vascular events by 25% in individuals without ASCVD (3), the remaining >70% incidence rate poses new clinical challenges as the residual ASCVD risk. The residual ASCVD risk includes hypertriglyceridemia and low high-density lipoprotein cholesterol (HDL-C) levels (5–9).

The assessment of hypertriglyceridemia as a risk factor for ASCVD has been shown to be more valuable in a non-fasting state than in a fasting state (6, 7, 10–13). Additionally, triglyceride (TG)-rich lipoproteins (TRLs) represented by chylomicron (CM) remnants, which increase with food intake, are not only the main cause of postprandial hypertriglyceridemia but are also considered to evoke atherosclerosis by deposition on the arterial intima (14). Furthermore, TRLs have also been reported to be independent risk factors for ASCVD (15, 16).

Currently, various drugs are available as therapeutic agents for hypertriglyceridemia. Especially fibrates and polyunsaturated fatty acids (PUFAs) have played a central role in pharmacotherapy for hypertriglyceridemia. Fibrates, which are peroxisome proliferator-activated receptor α (PPARα) agonists, can be expected to both decrease TG and increase HDL-C levels (17). Can fibrates prevent ASCVD? A meta-analysis conducted by Jun et al. revealed that fibrates reduced the risk of major cardiovascular events predominantly by prevention of coronary events (18). Meta-analyses published in a Cochrane review also demonstrated both primary and secondary preventive effects of fibrates on cardiovascular events, mainly due to their effects on coronary events (19, 20).

As a novel TG-lowering agent, pemafibrate, a selective PPARα modulator, has been launched in Japan. Compared with the conventional fibrates, PPARα agonists, pemafibrate has higher PPARα selectivity (21) and the same or better TRLs-lowering effects (22, 23). Additionally, a previous study examining the efficacy and safety of co-administration with statins showed that the frequencies of adverse events and adverse drug reactions with pemafibrate were comparable to those with placebo (24). Instead of conventional fibrates, pemafibrate is now expected to be more effective and safer therapeutic agent for patients with hypertriglyceridemia and low HDL-C levels.

Omega-3 fatty acid ethyl, the PUFA, is also able to decrease TG and slightly increase HDL-C levels. Clinical trials, including the JELIS trial conducted in Japan, have shown that treatment with eicosapentaenoic acid (EPA) in combination with statins reduces the ASCVD risk (25, 26). Therefore, EPA and docosahexaenoic acid (DHA) (EPA/DHA) are also expected to reduce the ASCVD risk under statin treatment. However, neither the STRENGTH trial, which examined the effects of EPA/DHA on cardiovascular outcomes, nor the ASCEND trial and the ORIGIN trial, which examined the efficacy of EPA/DHA on cardiovascular events in participants including those taking statins were able to demonstrate the benefit of EPA/DHA (27–29). Accordingly, the protective effects of n-3 PUFAs on cardiovascular events have not yet been well clarified.

Fasting apolipoprotein B-48 (apoB-48) levels have been reported to correlate with the incremental area under the curve (AUC) of TG levels after the high-fat meal ingestion, suggesting that the fasting apoB-48 level is a useful indicator for postprandial hypertriglyceridemia (30). Additionally, fasting apoB-48 level is correlated with carotid intima-media thickness (IMT) (31) and the CAD prevalence (32). Based on the above, fasting apoB-48 is considered to be a useful marker for ASCVD risk.

Reduction of the residual ASCVD risk under statin treatment is an urgent clinical challenge. Although a variety of TG-lowering agents are available, the evidence regarding which agents should be combined with statins in reducing residual ASCVD risk is inadequate. Both pemafibrate and omega-3 fatty acid ethyl are expected to have anti-atherosclerotic effects as therapeutic agents for hypertriglyceridemia, however, the differences between the two agents, especially in the lowering effects on TRLs, has not been verified.

To provide new evidence regarding pharmacotherapeutic options for postprandial hypertriglyceridemia, which is one of the residual risk factors for ASCVD under statin treatment, we designed this prospective, multicenter, open-label, randomized, parallel group trial. In the present study, we employed fasting apoB-48 level as a surrogate marker that reflects postprandial hypertriglyceridemia and prospectively compared the lowering effects of pemafibrate and omega-3 fatty acid ethyl on fasting apoB-48 levels in patients with dyslipidemia.

## 2. Materials and methods

### 2.1. Study design

The pemafibrate reduction of TRLs compared with omega-3 fatty acid ethyl for unmet needs in dyslipidemic patients on target to apoB-48 (PROUD48) study was a prospective, multicenter, open-label, randomized, parallel group, comparative trial to compare the effects of pemafibrate and omega-3 fatty acid ethyl on the fasting apoB-48 level, a surrogate marker for postprandial hypertriglyceridemia, in patients with dyslipidemia. The details of the rationale, design and protocol were previously described (33). This study was registered in the Japan Registry of Clinical Trials (jRCT) on the 28th of April 2020 (No. jRCTs071200011).

### 2.2. Study participants

This study was performed in Japanese patients with dyslipidemia in accordance with the principles of the Declaration of Helsinki and its amendments. The participants were ambulatory patients who presented to Asahikawa Medical University Hospital (Asahikawa, Hokkaido), Caress Sapporo Hokko Memorial Clinic (Sapporo, Hokkaido), Hiramitsu Heart Clinic (Nagoya, Aichi, Japan) and Keiyukai Yoshida Hospital (Asahikawa, Hokkaido). The participants were recruited between April 2020 and April 2021. Written informed consent for participation was obtained from all participants after the investigators have explained the study in full. The inclusion criteria were ambulatory patients with dyslipidemia receiving statin treatment for more than 4 weeks, with fasting TG levels of ≥177 mg/dl (2 mmol/L); aged 20–79 years; and those who provided written informed consent. The exclusion criteria were fasting TG levels ≥500 mg/dl (5.7 mmol/L); diabetic patients with HbA1c levels ≥9% and who need insulin treatment; type 1 diabetes; serum creatinine levels ≥1.5 mg/dl or higher; patients who used fibrates and nicotinic acids within 4 weeks; patients who used PUFAs including supplements within 24 weeks; symptomatic cardiovascular and cerebrovascular disorders; severe infections; acute hepatitis or liver cirrhosis; cancer; patients before or after surgery; women with pregnancy or during breastfeeding; patients who need lipid management with proprotein convertase subtilisin/kexin type 9 (PCSK9) inhibitors or microsomal triglyceride transfer protein (MTP) inhibitors; patients who have contraindications for pemafibrate and omega-3 fatty acid ethyl. The fasting TG level of ≥177 mg/dl in the inclusion criteria was set according to the fasting TG level of ≥150 mg/dl in the diagnostic criteria for dyslipidemia in the guidelines of the Japan Atherosclerosis Society (34) and considering the non-fasting TG level of ≥2 mmol/L in the joint consensus statement of the European Atherosclerosis Society and European Federation of Clinical Chemistry and Laboratory Medicine (35). The fasting TG level of ≥500 mg/dl (5.7 mmol/L) in the exclusion criteria was based on the levels commonly regarded as indicating severe hypertriglyceridemia (36, 37). Fasting in this study complied with the guidelines of the Japan Atherosclerosis Society of fasting for at least 10 h (34). A serum creatinine level ≥1.5 mg/dl as the exclusion criterion for renal dysfunction was based on the description in the package insert of pemafibrate as a criterion for patients with renal impairment requiring careful administration. The study protocol was approved by the Certified Review Board of the University of the Ryukyus for Clinical Research Ethics (No. CRB7200001) on 26 February 2020.

### 2.3. Randomization and intervention

Enrollment and randomization were performed centrally using an electronic data capture (EDC) system. After consent and enrollment, the participants were randomly allocated to the pemafibrate treatment group (PEMA) or omega-3 fatty acid ethyl treatment group (OMEGA-3) in a 1:1 ratio. This allocation was stratified based on three factors: fasting TG level (<300 or ≥300 mg/dl), sex (male or female), and age (<65 or ≥65 years). Participants allocated to the PEMA were given pemafibrate at a dose of 0.2 mg orally twice a day for 16 weeks with continuing statin treatment. Participants allocated to the OMEGA-3 were given omega-3 fatty acid ethyl at a dose of 2 g orally twice a day for 16 weeks with continuing statin treatment, as described previously (33). In principle, the addition of new drugs, discontinuation, or dose changes of all drugs including statins, pemafibrate and omega-3 fatty acid ethyl, were not permitted during the trial. Drugs that are contraindicated for co-administration with pemafibrate or omega-3 fatty acid ethyl were cyclosporine and rifampicin (contraindication for pemafibrate). Drugs that should be prohibited for use during the study due to the nature of the trial were insulin, fibrates, EPA, PCSK9 inhibitors and MTP inhibitors. All participants were on a diet with an optimized total energy intake based on their ideal body weight and daily activity to maintain an appropriate body weight during the study based on the Japan Atherosclerosis Society guidelines (34).

### 2.4. Endpoints

The primary endpoint was the percentage change in fasting apoB-48 level from baseline to 16 weeks. The key secondary endpoints included the change in fasting apoB-48 level from baseline to 16 weeks, the percentage changes in clinical variables from baseline to 16 weeks and the incidences of adverse events and diseases. Other secondary endpoints included the changes in clinical variables from baseline to 16 weeks. The clinical variables included parameters related to lipids and atherosclerosis (remnant lipoprotein cholesterol, small dense LDL-C, total cholesterol, TGs, LDL-C, HDL-C, apoA-I, apoA-II, apoB, apoC-II, apoC-III, apoE, and lipoprotein subfractions), parameters related to glycemic control [fasting plasma glucose, fasting immunoreactive insulin (IRI), homeostasis model assessment insulin resistance (HOMA-IR), and beta-cell function (HOMA-β) and HbA1c], blood biochemical parameters [aspartate aminotransferase (AST), alanine aminotransferase (ALT), gamma-glutamyl transpeptidase (γ-GTP), alkaline phosphatase (ALP), creatinine, estimated glomerular filtration rate (eGFR), and creatine kinase (CK)], fibrinogen and physical findings [blood pressure, body weight, body mass index (BMI), and waist circumference]. ApoB-48, remnant lipoprotein cholesterol, small dense LDL, total cholesterol, TGs, LDL-C, HDL-C, apoA-I, apoA-II, apoB, apoC-II, apoC-III, apoE, IRI, and HbA1c were measured at a central clinical laboratory (SRL, Hachioji, Japan). Serum apoB-48 concentrations were analyzed by a chemiluminescent enzyme immunoassay (38). Other clinical parameters were measured at each institution. Lipoprotein subfractions were analyzed by gel permeation high-performance liquid chromatography (GP-HPLC) at Skylight Biotech (Akita, Japan) to assess the sizes and concentrations of serum lipoproteins.

### 2.5. Data management

Clinical data in this study were managed with anonymized study-specific identification numbers using an EDC system. All data were managed by an independent data center (Nexis, Fukuoka, Japan), including entry, coding, security, storage and cleaning. Investigators and the head of each institution archive the information related to the study for 5 years from the date of study completion. The data center also archives the information related to this study, including electronic media, on the EDC system.

### 2.6. Sample size

This study compared the lowering effects of pemafibrate and omega-3 fatty acid ethyl on the percentage change in fasting apoB-48 level from baseline to 16 weeks. Previous reports showed that the percentage changes in fasting apoB-48 level on treatment with pemafibrate at 0.4 mg/day or omega-3 fatty acid ethyl at 4 g/day combined with a statin were −57.3 ± 24.2% (24) and −22.0% (39), respectively. We employed 58.2% as the standard deviation (SD) for the percentage change in fasting apoB-48 level on treatment with 4 g/day omega-3 fatty acid ethyl monotherapy in a pilot study by Hiramitsu et al. reported in Japanese in 2013, since no study has been reported in English gave the SD for combined treatment with omega-3 fatty acid ethyl and a statin. Based on these parameters and with a two-sided alpha of 0.05 and power of 0.8, 44 participants per group were required to detect a significant difference in the percentage change between the two groups. Considering a dropout rate of 10%, the target number of total participants was set to 100. However, we reset the total target number of participants to 128 due to the COVID-19 pandemic, which may increase the dropout rate compared with our original estimation, as previously described (33).

### 2.7. Statistical analysis

No interim analysis was performed. The primary and secondary endpoints except for adverse events were analyzed primarily using the full analysis set (FAS) and secondary per protocol set (PPS). The FAS included participants enrolled in this study and assigned to treatment groups excluding those with major study protocol violations, such as failing to obtain consent or enrollment outside the study period. The PPS included participants from the FAS after excluding those who violated the inclusion or exclusion criteria, used drugs contraindicated for pemafibrate and omega-3 fatty acid ethyl or adhered poorly to pemafibrate and omega-3 fatty acid ethyl (<75% or ≥120%) during the study. The medication adherence rate was calculated as the followings: 100 × frequency of taking medication since last visit/assigned frequency of medication since last visit, by asking the participants about the actual frequencies of medication since the last visit. As a secondary endpoint, adverse events were analyzed using the safety analysis set, which includes participants who received an assigned treatment at least once. Continuous and categorical variables were presented as means ± SDs or medians [interquartile ranges (IQR) or min-max values (Min-Max)] and as frequencies with percentages, respectively.

Originally, we planned to use the unpaired *t* test to compare the primary endpoint, the percentage change in fasting apoB-48 level from baseline to 16 weeks, between the two groups (33). However, since the obtained data on the percentage change in fasting apoB-48 level did not show the normality, the primary endpoint was compared using Mann–Whitney *U* test. The secondary endpoints were analyzed using the paired *t* test or Wilcoxon’s signed-rank test for within-group comparisons, the unpaired *t* test or Mann–Whitney *U* test for comparisons between two groups. The baseline characteristics of the participants in the two groups were compared using the chi-square test or Fisher’s exact test for categorical variables and *t* test or Mann–Whitney *U* test for continuous variables. All *P*-values were two-sided with *P* < 0.05 taken to indicate statistical significance. All statistical analyses were performed by the study statistician (M. Sakurai) at the data center (Nexis, Fukuoka, Japan) using SPSS ver. 26 (IBM Corp., Armonk, NY, USA).

## 3. Results

### 3.1. Participants and baseline characteristics

**FIGURE 1:**
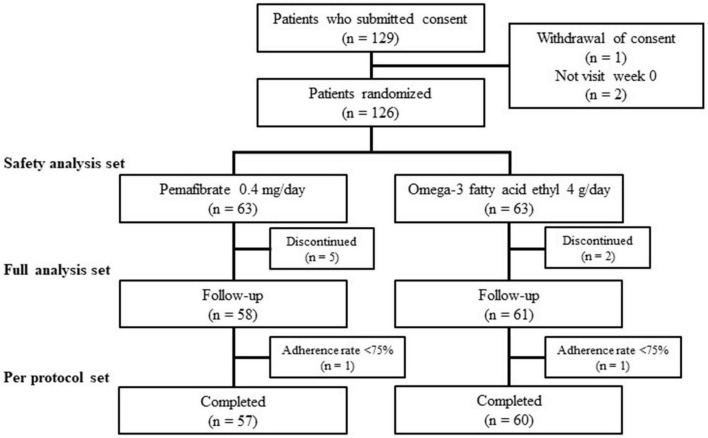
Flow chart of participant recruitment.

A total of 129 participants were recruited and assessed for eligibility. Three participants were excluded because of withdrawal of consent and failure to visit. The remaining 126 participants were randomly assigned to the PEMA (*n* = 63) and OMEGA-3 (*n* = 63) and were included in the SAS. Then, 58 participants in PEMA and 61 participants in OMEGA-3, excluding those who discontinued the treatment, were followed-up and included in the FAS. Finally, 57 participants in PEMA and 60 participants in OMEGA-3 completed the follow-up by 22 May 2021 and were included in the PPS (Figure 1).

Table 1 shows the baseline characteristics of the participants. Baseline characteristics were well balanced between the two groups, except for the prevalence of hypertension, the mean apoC-II level, and the median alkaline phosphatase level. The median apoB-48 level was 6.7 and 6.4 μg/ml in PEMA and OMEGA-3, respectively. The median TG level was 196 and 218 mg/dl in PEMA and OMEGA-3, respectively. Fibrinogen values were not sufficiently obtained in both groups.

**TABLE 1:** Baseline characteristics of participants.

	All		PEMA		OMEGA-3		*P*
	*n*		*n*		*n*		
Age (years)	119	63.6 ± 9.7	58	64.4 ± 8.6	61	62.8 ± 10.6	0.362
Sex, female, n (%)	119	48(38.7)	58	20(34.5)	61	26(42.6)	0.362
Diabetes mellitus, n (%)	119	56(47.0)	58	25(43.1)	61	31(50.8)	0.399
Hypertension, n (%)	119	83(69.7)	58	47(81.0)	61	36(59.0)	0.009
Diabetic nephropathy, n (%)	119	36(30.3)	58	13(22.4)	61	23(37.7)	0.058
Diabetic retinopathy, n (%)	119	3(2.5)	58	1(1.7)	61	2(3.3)	0.580
Diabetic neuropathy, n (%)	119	1(0.8)	58	0(0)	61	1(1.6)	0.324
Kidney disease, n (%)	119	35(29.4)	58	14(24.1)	61	21(34.4)	0.194
Liver disease, n (%)	119	51(42.0)	58	23(37.9)	61	28(45.9)	0.332
Cerebrovascular disease, n (%)	119	9(7.6)	58	7(12.1)	61	2(3.3)	0.073
Cardiac disease, n (%)	119	37(31.1)	58	17(29.3)	61	20(32.8)	0.638
History of Cerebrovascular disease, n (%)	119	10(8.4)	58	7(12.1)	61	3(4.9)	0.074
History of Cardiac disease, n (%)	119	33(27.7)	58	14(24.1)	61	19(31.1)	0.617
Habitual alcohol intake_yes, n (%)	119	73(61.3)	58	34(58.6)	61	39(63.9)	0.552
Never smoker, n (%)	119	44(37.0)	58	19(32.8)	61	25(41.0)	0.399
Ex-smoker, n (%)	119	46(38.7)	58	26(44.8)	61	20(32.8)	
Current-smoker, n (%)	119	29(24.3)	58	13(22.4)	61	16(26.2)	
Body weight (kg)	119	70.1 ± 13.9	58	70.4 ± 12.3	61	69.9 ± 15.4	0.839
Waist circumference (cm)	118	93.3 ± 9.5	57	94.3 ± 8.7	61	92.6 ± 10.2	0.293
BMI (kg/m^2^)	119	26.4 ± 4.0	58	26.7 ± 3.3	61	26.2 ± 4.6	0.494
SBP (mmHg)	118	130.0 ± 8.1	57	130.1 ± 8.3	61	130.1 ± 8.4	0.948
DBP (mmHg)	118	69.9 ± 8.5	57	71.2 ± 9.2	61	69.1 ± 8.9	0.199
Total cholesterol (mg/dl)	119	182.0 ± 25.2	58	182.3 ± 23.7	61	181.6 ± 26.9	0.880
Triglycerides (mg/dl)	119	206(71–492)	58	196(76–380)	61	218(71–492)	0.053
HDL-C (mg/dl)	119	49.7 ± 11.6	58	50.5 ± 12.3	61	48.9 ± 10.8	0.439
LDL-C (mg/dl)	119	97.0 ± 23.3	58	97.6 ± 24.3	61	96.4 ± 22.4	0.787
Small dense LDL (mg/dl)	119	37.3 ± 13.4	58	35.9 ± 12.9	61	38.6 ± 13.8	0.258
Remnant lipoprotein cholesterol (mg/dl)	119	8.7 ± 3.8	58	8.1 ± 3.1	61	9.2 ± 4.4	0.121
ApoA-I (mg/dl)	119	150.7 ± 25.9	58	152.7 ± 27.9	61	148.8 ± 24.0	0.415
ApoA-II (mg/dl)	119	30.9 ± 4.7	58	31.2 ± 4.4	61	30.6 ± 4.9	0.502
ApoB (mg/dl)	119	89.8 ± 16.9	58	89.2 ± 17.1	61	90.3 ± 16.9	0.744
ApoB-48 (μg/ml)	119	6.5(1.5–40)	58	6.7(1.5–40)	61	6.4(2.8–40)	0.938
ApoC-II (mg/dl)	119	5.9 ± 1.7	58	5.5 ± 1.3	61	6.3 ± 2.0	0.009
ApoC-III (mg/dl)	119	14.2 ± 3.7	58	13.5 ± 2.9	61	14.8 ± 4.2	0.065
ApoE (mg/dl)	119	4.8 ± 1.3	58	4.7 ± 1.2	61	4.8 ± 1.3	0.912
Fasting plasma glucose (mg/dl)	119	118.9 ± 26.8	58	120.9 ± 31.6	61	116.9 ± 21.4	0.431
HbA1c (%)	119	6.2 ± 0.9	58	6.2 ± 0.9	61	6.2 ± 0.8	0.842
Insulin (IU/l)	119	9.02(1.63–110)	58	9.12(2.59–110)	61	9.02(1.63–32.0)	0.582
HOMA-IR	119	2.54(0.39–39.4)	58	2.34(0.74–39.4)	61	2.68(0.39–7.38)	0.553
HOMA-β (%)	119	63.6(9.6–500.9)	58	60.3(9.6–604.6)	61	66.3(12.3–500.9)	0.963
AST (IU/l)	119	25(13–110)	58	25(13–110)	61	25(13–97)	0.414
ALT (IU/l)	119	28(10–174)	58	29(13–162)	61	25(10–174)	0.283
γ-GTP (IU/l)	119	40(11–545)	58	43(11–431)	61	37(12–545)	0.439
ALP (IU/l)	119	222(24–462)	58	205(24–340)	61	227(48–462)	0.001
CK (IU/l)	119	100(37–417)	58	101(37–417)	61	99(42–380)	0.248
Cr (mg/dl)	119	0.82 ± 0.20	58	0.81 ± 0.21	61	0.83 ± 0.20	0.519
eGFR (ml/min/1.73 m^2^)	119	69.5 ± 16.5	58	71.4 ± 17.1	61	67.6 ± 15.7	0.208
Fibrinogen (mg/dl)	39	293.4 ± 45.9	21	285.3 ± 41.5	18	302.8 ± 50.1	0.239

The data are presented as n (%) or medians (Min-Max) or means ± SDs. *P* values are for comparison between the two groups. BMI, body mass index; SBP, systolic blood pressure; DBP, diastolic blood pressure; HDL-C, high-density lipoprotein cholesterol; LDL-C, low-density lipoprotein cholesterol; Apo, apolipoprotein; HOMA-IR, homeostasis model assessment insulin resistance; HOMA-β, homeostasis model assessment beta-cell function; AST, aspartate aminotransferase; ALT, alanine aminotransferase; γ-GTP, gamma-glutamyl transpeptidase; ALP, alkaline phosphatase; CK, creatine kinase; eGFR, estimated glomerular filtration rate.

### 3.2. Primary endpoint

The percentage changes in fasting apoB-48 level from baseline to week 16 in PEMA and OMEGA-3 were −50.8% (IQR −62.9 to −30.3%) and −17.5% (IQR −38.3 to 15.3%), respectively. The difference between the two groups was statistically significant (*P* < 0.001) (Table 2).

**TABLE 2:** Percentage change in fasting apoB-48 level from baseline to week 16.

	PEMA (*n* = 58)	OMEGA-3 (*n* = 61)	*P*
Median	−50.8	−17.5	<0.001
IQR	−62.9 to -30.3	−38.3 to 15.3	
95% CI	−85.2 to 9.65	−59.9 to 110.5	
Min-Max	−89.5 to 18.4	−71.7 to 455.1	

The data are presented as medians. *P* value is for comparison between the two groups. IQR, interquartile range. 95% CI, 95% confidence interval.

### 3.3. Key secondary endpoints

The changes in fasting apoB-48 level from baseline to week 16 in PEMA and OMEGA-3 were −3.10 μg/ml (IQR −5.63 to −1.87 μg/ml) and −0.90 μg/ml (IQR −2.95 to 0.65 μg/ml), respectively. This difference was also statistically significant (*P* < 0.001) (Table 3).

**TABLE 3:** Change in fasting apoB-48 level (μg/ml) from baseline to week 16.

	PEMA (*n* = 58)	OMEGA-3 (*n* = 61)	*P*
Median	−3.10	−0.90	<0.001
IQR	−5.63 to −1.87	−2.95 to 0.65	
95% CI	−12.52 to −0.42	−6.67 to 11.65	
Min-Max	−17.3 to 1.20	−24.8 to 18.6	

The data are presented as medians. *P* value is for comparison between the two groups. IQR, interquartile range; CI, confidence interval.

Greater decreases with significant differences in the percentage changes from baseline to week 16 in TG, remnant lipoprotein cholesterol, apoC-III, fasting plasma glucose, ALT, γ-GTP, and ALP levels were observed in PEMA, compared with OMEGA-3. Also, greater increases with significant differences in those in HDL-C, apoA-I and apoA-II levels were observed in PEMA, compared with OMEGA-3. On the other hand, greater decreases with significant differences in those in total cholesterol, LDL-C and apoB levels were observed in OMEGA-3, compared with PEMA. In addition, compared with OMEGA-3, greater increase with significant difference in the percentage change from baseline to week 16 in creatinine level was observed in PEMA. In association with the result, greater decrease in that in eGFR level was also observed in PEMA, compared with OMEGA-3 (Table 4).

**TABLE 4:** Percentage changes in clinical variables from baseline to week 16.

	PEMA (*n* = 58)		OMEGA-3 (*n* = 61)		*P*
	Mean	SD	Mean	SD	
Body weight	0.00	3.45	0.11	2.74	0.846
Waist circumference	-0.31	4.24	0.04	5.44	0.708
BMI	0.00	3.45	0.11	2.74	0.841
SBP	-0.55	7.45	0.12	7.62	0.635
DBP	4.34	22.88	4.01	12.36	0.921
Total cholesterol	1.8	14.8	-6.1	10.5	0.001
Triglycerides	-38.3	26.8	-24.7	28.6	0.009
HDL-C	14.5	21.3	5.6	18.2	0.016
LDL-C	14.3	27.3	-9.0	16.5	0.000
Small dense LDL	-15.6	29.2	-14.1	21.0	0.753
Remnant lipoprotein cholesterol	-46.7	28.9	-28.9	40.0	0.007
ApoA-I	4.8	15.1	-1.1	10.1	0.013
ApoA-II	46.6	23.4	-5.7	11.2	<0.001
ApoB	-0.8	17.7	-7.9	12.0	0.012
ApoC-II	-18.7	23.7	-16.9	14.7	0.623
ApoC-III	-32.2	15.8	-11.1	15.5	0.000
ApoE	-7.4	18.7	-7.2	16.7	0.942
Fasting plasma glucose	-3.7	13.1	11.0	26.3	<0.001
HbA1c	1.2	8.6	3.1	10.8	0.314
Insulin	15.7	136.6	61.9	274.0	0.250
HOMA-IR	17.9	172.8	109.1	518.8	0.206
HOMA-β	24.7	119.0	20.3	123.3	0.843
AST	11.0	37.5	10.4	27.7	0.923
ALT	-11.8	42.0	17.1	40.2	<0.001
γ-GTP	-37.1	41.6	12.4	69.0	<0.001
ALP	-35.1	23.4	-9.7	21.2	<0.001
CK	9.9	77.8	15.5	55.6	0.649
Cr	3.9	10.6	-0.7	9.8	0.014
eGFR	-3.2	9.5	1.9	10.8	0.008
(Fibrinogen)	-18.9	15.4	-0.98	10.9	0.001

The data are presented as means ± SDs. *P* values are for comparison between the two groups. BMI, body mass index; SBP, systolic blood pressure; DBP, diastolic blood pressure; HDL-C, high-density lipoprotein cholesterol; LDL-C, low-density lipoprotein cholesterol; Apo, apolipoprotein; HOMA-IR, homeostasis model assessment insulin resistance; HOMA-β, homeostasis model assessment beta-cell function; AST, aspartate aminotransferase; ALT, alanine aminotransferase; γ-GTP, gamma-glutamyl transpeptidase; ALP, alkaline phosphatase; CK, creatine kinase; eGFR, estimated glomerular filtration rate.

In GP-HPLC analyses, the percentage change in cholesterol content in LDL particles from baseline to week 16 in PEMA was significantly reduced in the very small LDL subclass and was significantly increased in the large and medium LDL subclasses, compared with OMEGA-3. The GP-HPLC analyses also showed that the percentage change in cholesterol content in HDL particles from baseline to week 16 in PEMA was significantly increased in the medium, small and very small HDL subclasses and was significantly reduced in the very large and large HDL subclasses, compared with OMEGA-3 (Table 5).

**TABLE 5:** Percentage changes in cholesterol content by GP-HPLC analysis from baseline to week 16.

		PEMA (n = 58)	OMEGA-3 (n = 61)	*P*
Subclass	Particle diameter (nm)	Median (Min-Max)	Median (Min-Max)	
CM	>90	−69.8(−96.3 to 33.2)	−55.7(−93.8 to 48.1)	0.009
	75	−58.7(−93.8 to 62.8)	−45.9(−89.9 to 41.4)	0.019
L-VLDL	64	−49.0(−87.1 to 90.5)	−38.4(−82.8 to 45.4)	0.016
	53.6	−49.0(−86.9 to 76.8)	−28.6(−74.7 to 59.4)	<0.001
	44.5	−17.2(−69.7 to 111.1)	−12.7(−64.0 to 41.7)	0.450
M-VLDL	36.8	−30.9(−79.1 to 76.3)	−11.3(−55.1 to 59.7)	0.001
S-VLDL	31.3	31.5(−47.8 to 445.0)	−12.5(−55.0 to 108.6)	<0.001
L-LDL	28.6	30.9(−30.0 to 449.1)	−5.9(−35.9 to 137.9)	<0.001
M-LDL	25.5	14.7(−42.7 to 111.9)	−1.6(−44.1 to 86.5)	0.002
S-LDL	23.0	4.5(−61.9 to 109.7)	0.2(−45.5 to 162.5)	0.669
VS-LDL	20.7	7.3(−64.9 to 123.7)	3.9(−46.1 to 162.5)	0.770
	18.6	−3.4(−54.2 to 79.0)	12.0(−52.2 to 125.8)	0.022
	16.7	−1.7(−54.9 to 66.9)	8.1(−29.1 to 102.4)	0.001
VL-HDL	15.0	−4.2(−69.4 to 71.8)	8.9(−56.6 to 120.0)	0.003
	13.5	−41.9(−83.0 to 67.5)	17.5(−55.7 to 221.5)	<0.001
L-HDL	12.1	−6.3(−67.6 to 106.3)	23.4(−43.7 to 163.4)	<0.001
M-HDL	10.9	45.0(−17.5 to 120.6)	7.7(−30.6 to 73.8)	<0.001
S-HDL	9.8	45.2(−15.6 to 137.2)	−3.0(−37.4 to 52.1)	<0.001
VS-HDL	8.8	47.4(−9.5 to 132.6)	−0.7(−30.1 to 56.4)	<0.001
	7.6	22.5(−12.7 to 97.8)	2.8(−15.7 to 44.2)	<0.001

The data are presented as medians (Min-Max). *P* values are for comparison between the two groups. Lipoprotein subclasses are listed in descending order of particle diameter from CM to VS-HDL. CM, chylomicron; L-VLDL, large very low-density lipoprotein; M-VLDL, medium very low-density lipoprotein; S-VLDL, small very low-density lipoprotein; L-LDL, large low-density lipoprotein; M-LDL, medium low-density lipoprotein; S-LDL, small low-density lipoprotein; VS-LDL, very small low-density lipoprotein; VL-HDL, very large high-density lipoprotein; L-HDL, large high-density lipoprotein; M-HDL, medium high-density lipoprotein; S-HDL, small high-density lipoprotein; VS-HDL, very small high-density lipoprotein.

### 3.4. Other secondary endpoints

The other secondary endpoints, the changes in clinical variables from baseline to week 16, including within-group comparisons, were summarized in Supplementary Tables 1, 2. From baseline to week 16, apoB-48 levels were significantly decreased in both PEMA (6.7 μg/ml [Min-Max 1.5–40.0 μg/ml] to 3.3 μg/ml [0.8–22.7 μg/ml], *P* < 0.001) and OMEGA-3 (6.4 μg/ml [Min-Max 2.80–40.0 μg/ml] to 5.7 μg/ml [1.7–36.5 μg/ml], *P* = 0.026). The changes in fasting apoB-48 level from baseline to week 16 in PEMA and OMEGA-3 are as described above and Table 3. TG levels also decreased significantly in both PEMA (196 mg/dl [Min-Max 76–380 mg/dl] to 112 mg/dl [59–340 mg/dl], *P* < 0.001) and OMEGA-3 (218 mg/dl [Min-Max 71–492 mg/dl] to 153 mg/dl [54–475 mg/dl], *P* < 0.001) from baseline to week 16. The mean changes in TG level from baseline to week 16 in PEMA and OMEGA-3 were –83.34 ± 63.53 mg/dl and –62.21 ± 73.35 mg/dl, respectively, and there were no significant differences between the two groups (*P* = 0.096). Others are also detailed in Supplementary Tables 1, 2.

### 3.5. Adverse events

As shown in Table 6, adverse events occurred in 9 of 63 (14.3%) patients in PEMA and 3 of 63 (4.8%) patients in OMEGA-3. Drug-related and drug-unrelated adverse events requiring discontinuation of treatment were reported only in PEMA (9 cases in 3 patients). Adverse events not requiring discontinuation of treatment were reported in 6 cases (6 patients) in PEMA, and in 4 cases (3 patients) in OMEGA-3. Serious adverse events associated with drug were not observed in either group.

**TABLE 6:** Adverse events.

	PEMA (*n* = 63)	OMEGA-3 (*n* = 63)
AEs	9 (14.3)	3 (4.8)
Participants who discontinued treatment because of drug-related AE		
Nausea	1 (1.6)	0 (0.0)
Pain in the extremities	1 (1.6)	0 (0.0)
Erythema on the forearm	1 (1.6)	0 (0.0)
Itching in the forearm	1 (1.6)	0 (0.0)
General malaise	1 (1.6)	0 (0.0)
Anorexia	1 (1.6)	0 (0.0)
Participants who discontinued treatment because of drug-unrelated AE		
Neck stiffness	1 (1.6)	0 (0.0)
Tooth pain	1 (1.6)	0 (0.0)
Sepsis associated with enteritis	1 (1.6)	0 (0.0)
Other AEs		
Worsening of glycemic control	2 (3.2)	1 (1.6)
Acute epiglottitis	1 (1.6)	0 (0.0)
Dental caries	1 (1.6)	0 (0.0)
Liver dysfunction	1 (1.6)	0 (0.0)
Muscular pain	1 (1.6)	0 (0.0)
Fall	0 (0.0)	1 (1.6)
Lumbago	0 (0.0)	1 (1.6)
Elevation of serum CK	0 (0.0)	1 (1.6)

The data are presented as n (%). AE, adverse event; CK, creatine kinase.

## 4. Discussion

To the best of our knowledge, this is the first randomized trial to directly compare the lowering effects of pemafibrate and omega-3 fatty acid ethyl on fasting apoB-48, a marker reflecting postprandial hypertriglyceridemia, which is one of the residual risks for ASCVD with statin treatment. In the present study, the percentage changes in fasting apoB-48 level from baseline to week 16 were −50.8 and −17.5%, respectively, and the decrease was significantly greater in PEMA, compared with OMEGA-3. These findings demonstrated that pemafibrate is superior to omega-3 fatty acid ethyl in lowering effect of fasting apoB-48, and also provide the important insights regarding which drug, pemafibrate or omega-3 fatty acid ethyl, is the better option for the treatment of hypertriglyceridemia to reduce the residual ASCVD risk associated with TRLs in patients with dyslipidemia receiving statin treatment. The decreases in percentage changes in fasting apoB-48 levels by pemafibrate and omega-3 fatty acid ethyl were similar to the previous studies conducted with different treatment periods (24, 39). Therefore, we also demonstrated that the apoB-48-lowering effects of pemafibrate and omega-3 fatty acid ethyl are reproducible in dyslipidemic patients with statin treatment.

There are no previous reports verifying how much reduction in apoB-48 leads to a reduction in the residual ASCVD risk. Therefore, it is not easy to discuss the extent to which the reduction in apoB-48 level observed in this study has clinical impact in reducing residual ASCVD risk in statin-treated patients with dyslipidemia. However, there are several reports examining the close relationship between apoB-48 and ASCVD. Nakatani et al. demonstrated that fasting apoB-48 levels positively correlated with IMT of the carotid artery and fasting apoB-48 level was an independent determinant of carotid IMT in subjects with normal TG levels (31). Alipour et al. also showed that fasting apoB-48 levels positively correlated with carotid IMT in patients with atherosclerotic risk factors (40). A recent case-control study conducted by Tian et al. showed the relationship between fasting apoB-48 levels and large artery atherosclerotic stroke (41). Additionally, Masuda and colleagues revealed the positive correlation between fasting apoB-48 levels and the prevalence of CAD and also proposed an apoB-48 level of 4.34 μg/ml as the optimal cut-off value for CAD versus non-CAD by receiver-operating characteristic curve analysis (32). Moreover, Mori et al. showed that fasting apoB-48 levels were higher in patients with not only chronic CAD but also new-onset CAD than those with non-CAD, independent of LDL-C levels, and the increase in apoB-48 levels was associated with progression of coronary artery lesions in CAD patients received percutaneous coronary intervention and LDL-C lowering therapy (42). Based on these reports, it is expected that pharmacotherapy that lowers fasting apoB-48 levels will result in a reduction in residual ASCVD risk associated with TRLs. In particular, the results of the present study suggest that pemafibrate, compared with omega-3 fatty acid ethyl, is expected to more improve cardiovascular outcomes associated with TRLs-related residual ASCVD risk. However, we consider that prospective comparison trial with cardiovascular outcomes as primary endpoint is needed to clarify the differences in effects on cardiovascular outcomes associated with TRLs-related residual ASCVD risk between the two agents.

What are the possible mechanisms underlying the difference in apoB-48-lowering effect between the two agents? Increase in TG levels in the postprandial state is due to increased production of TRLs. TRLs include endogenous very low-density lipoprotein (VLDL) from the liver and exogenous CM from the small intestine. These TRLs and their remnants are thought to be involved individually in postprandial hypertriglyceridemia. However, Masuda et al. demonstrated that apoB-48-containing lipoproteins, but not apoB-100-containing lipoproteins were increased after high-fat meal ingestion with elevation of TG levels, suggesting that postprandial hypertriglyceridemia was mainly due to increased CM and CM remnants, but not VLDL (30). Fasting apoB-48 level used in this study is a useful indicator for postprandial hypertriglyceridemia. Considering the previous report by Masuda et al., the difference in apoB-48-lowering effect between pemafibrate and omega-3 fatty acid ethyl observed in this study may be involved in the difference in the effects of both agents on CM metabolism in the postprandial state.

Polyunsaturated fatty acids have been reported to show the TRLs-lowering effects by mainly decreasing VLDL production from the liver (43). Although the detailed mechanism of the TRLs-lowering effect of PUFAs via CM metabolism has not been fully elucidated, omega-3 fatty acid ethyl has been reported to decrease CM particle size by lowering levels of apoC-III, which suppresses lipoprotein lipase (LPL) activity (44).

Fibrates, PPARα agonists, have been reported to show the TRLs-lowering effects by predominantly decreasing VLDL production and secretion from the liver (45, 46). Similar to conventional fibrates, pemafibrate suppresses VLDL production and secretion from the liver by its selective PPARα agonism, which is considered to be the main mechanism of TRLs-lowering effect of pemafibrate (47). On the other hand, pemafibrate also has been shown to effectively suppress CM synthesis and secretion from the small intestine and increase clearance of CM remnants by its selective PPARα agonism as the following mechanisms: (1) decreased intestinal mRNA expression of ApoB and Npc1l1 (suppress CM synthesis and secretion) and (2) suppression of hepatic ApoC3 mRNA expression (increase clearance of CM remnants via increased LPL activity) (47, 48). Interestingly, the former was also observed in fenofibrate, but the latter was not (48). Accordingly, we speculate that these favorable effects of pemafibrate by selective PPARα agonism on CM metabolism, which is considered to be more effective than conventional fibrates, led to the obvious difference in apoB-48-lowering effect between pemafibrate and omega-3 fatty acid ethyl in this study. Furthermore, Duez et al. demonstrated that intestinal apoB-48-containing TRLs production was increased in hyperinsulinemic, insulin-resistant subjects (49). In the present study, although no difference in HOMA-IR was observed between the two treatment groups, HOMA-IR level was significantly decreased from baseline to week 16 in only PEMA. This result is very interesting because it suggests the possibility that the improvement of insulin resistance by pemafibrate may additionally contribute to the apoB-48-lowering effect other than its selective PPARα agonism.

The percentage changes in HDL-C level from baseline to week 16 were 14.5 and 5.6%, respectively, and the increase was significantly greater in PEMA, compared with OMEGA-3. Thus, the present study demonstrated that pemafibrate is superior to omega-3 fatty acid ethyl in not only the apoB-48-lowering effect but also the HDL-C increasing effect. Low HDL-C level has also been reported to be a residual ASCVD risk, along with hypertriglyceridemia in patients with dyslipidemia receiving statin treatment (5–9). We consider that the findings regarding the effect of pemafibrate on HDL-C also provide an additional insight in the selection of pharmacotherapy to reduce residual risk of ASCVD in dyslipidemic patients with statin treatment.

In this study, we also analyzed the changes in lipoprotein subfractions by GP-HPLC to elucidate which of the two agents shows a greater anti-atherosclerotic lipoprotein profile. Compared with OMEGA-3, PEMA resulted in a decrease in small LDL particles and an increase in small HDL particles. These findings indicate that pemafibrate is more anti-atherosclerotic than omega-3 fatty acid ethyl in its effect on lipoprotein profile. It is widely known that small dense LDL is closely associated with elevated ASCVD risk (50, 51). This is because small dense LDL is susceptible to oxidative degeneration, and oxidized LDL is involved in atherosclerotic processes such as vascular endothelial damage, increased monocyte infiltration into the vascular wall, and foam cell formation (52, 53). On the other hand, why is an increase in small HDL particles anti-atherosclerotic? Previous reports demonstrated that cholesterol efflux capacity (CEC) from macrophage, a metric of HDL function, correlated inversely with the presence of CAD and carotid IMT (54), and incidence of cardiovascular events (55). And smaller HDL subfractions were shown to have greater CEC (56). From these findings, an increase in small HDL particles in lipoprotein profile is considered to have anti-atherosclerotic effects. Therefore, pemafibrate, compared with omega-3 fatty acid ethyl, is expected to provide an additional residual ASCVD risk reduction in patients with dyslipidemia receiving statin treatment by showing a more anti-atherosclerotic lipoprotein profile such as a decrease in small LDL particles and an increase in small HDL particles, in addition to the apoB-48-lowering and HDL-C-increasing effects.

Pemafibrate treatment group showed a significant decrease in fasting plasma glucose levels after 16-week treatment, compared with OMEGA-3. As shown in Table 1, although there was no obvious difference in prevalence of diabetes mellitus between PEMA and OMEGA-3, approximately half of the study participants had diabetes mellitus. Reflecting that, participants of this study had a mean HbA1c levels of 6.2%, a mean fasting plasma glucose levels of 118.9 mg/dl, and a median HOMA-IR 2.54. Both glucose intolerance and insulin resistance have been shown to be conditions with high cardiovascular risk (57–60). Although no difference in HOMA-IR was observed between the two treatment groups, the significant reduction in fasting plasma glucose observed in PEMA compared with OMEGA-3 could be a favorable finding in considering the effect of pemafibrate on cardiovascular risk reduction.

Focusing on the changes from baseline to week 16, fasting plasma glucose level was significantly decreased in PEMA and significantly increased in OMEGA-3. HOMA-IR levels were significantly decreased in PEMA, but no change was observed in OMEGA-3 (Supplementary Table 1). These results not only suggested that pemafibrate was superior to omega-3 fatty acid ethyl in the effect on glucose metabolism, but also raised concerns that omega-3 fatty acid ethyl might have an unfavorable effect on glucose metabolism. Similar to our findings, previous studies reported that omega-3 fatty acid ethyl may adversely affect glucose tolerance (61, 62). However, based on the results of meta-analyses of older studies and more recent prospective studies including REDUCE-IT trial, it has been concluded that PUFAs do not adversely affect glucose metabolism (63). Therefore, it cannot be concluded that the increase in fasting plasma glucose levels observed in OMEGA-3 in this study is necessarily an unfavorable finding in TG-lowering therapy with omega-3 fatty acid ethyl.

Of course, hypertension is also an important risk factor for ASCVD (34). To our knowledge, the effect of pemafibrate on blood pressure remains to be determined. On the other hand, omega-3 fatty acid ethyl has been reported to reduce blood pressure, which was not observed in this study. One reason for this may be that the prevalence of hypertension was lower in OMEGA-3 than in PEMA. Additionally, a systematic review and meta-analysis conducted by Zhang et al. showed that the optimal dose of omega-3 fatty acid ethyl for blood pressure-lowering was between 2 g/day and 3 g/day (64). Since the dose of omega-3 fatty acid ethyl in this study was 4 g/day, this may also be the reason why no effect of omega-3 fatty acid ethyl on blood pressure was observed.

Interestingly, greater decreases with significant differences in the percentage changes from baseline to week 16 in ALT, γ-GTP, and ALP levels were observed in PEMA, compared with OMEGA-3. Previous study conducted by Nakajima and colleagues reported that pemafibrate significantly improved serum ALT, γ-GTP and ALP levels in patients with non-alcoholic fatty liver disease (NAFLD), compared with placebo (65). NAFLD is closely related to dyslipidemia, and also is newly recognized as a condition with increased ASCVD risk (66, 67). Considering the relationship between NAFLD and ASCVD risk, the improvement of liver function by pemafibrate may partially contribute to the reduction of ASCVD risk. However, we did not examine the prevalence of NAFLD in the study participants, and their median ALT, γ-GTP and ALP levels were within normal range. Therefore, our findings cannot be unconditionally applied to the discussion of NAFLD and ASCVD risk.

Compared with OMEGA-3, we observed a significant increase in creatinine levels and a significant decrease in eGFR levels in PEMA after 16-week treatment. Similar findings were observed in a phase 3 trial of pemafibrate (K-877) in comparison with fenofibrate in Japan (22). However, it has been considered that these findings in PEMA would be essentially harmless, similar to the conventional fibrates, because of the following reasons: (1) not associated with decrease in GFR estimated by inulin clearance and (2) reversible after cessation (22). In the FIELD study, which examined the effects of fenofibrate on renal function in patients with type 2 diabetes, Davis and colleagues discussed the possible cause of the elevated creatinine levels by fenofibrate as follows: (1) increased muscle production of creatinine; (2) changes in tubular creatinine secretion; (3) reduced glomerular function; and (4) altered renal plasma flow (68). Also, the elevation of creatinine levels is considered to be transient. Although we believe that the effects of pemafibrate on renal function are not expected to be serious, restrictions on co-administered drugs and their doses may be of clinical concern.

No serious drug-related adverse events were observed in either treatment group, however, adverse events reported in PEMA were approximately three times as many as in OMEGA-3. Therefore, longer-term administration of pemafibrate may require careful observation for the occurrence of adverse events.

From baseline to week 16, we observed a significant increase in LDL-C levels in PEMA and a significant decrease in LDL-C levels in OMEGA-3. In addition, greater decrease with significant difference in the percentage change from baseline to week 16 in LDL-C level was observed in OMEGA-3, compared with PEMA. As shown in previous report (69), we consider that the increase in LDL-C levels in PEMA is probably related to the increased efficiency of conversion of TRL remnants to LDL by pemafibrate. Furthermore, we also speculate that this finding reflects an increase in buoyant LDL particles, which has been reported to have a lower ASCVD risk, as a result of a decrease in small dense LDL (70). However, there is also concern that the increased LDL-C levels observed in PEMA may adversely affect the lowering effect of pemafibrate on residual ASCVD risk associated with TRLs. On the other hand, it is somewhat difficult to discuss why decreased LDL-C levels were observed in OMEGA-3. In general, omega-3 fatty acid ethyl is not expected to decrease LDL-C level. A systematic review and meta-analysis conducted by Yang et al. showed that although the monotherapy of omega-3 fatty acid ethyl resulted in increased LDL-C levels, combined therapy with omega-3 fatty acid ethyl and statin did not change the LDL-C levels (71). Although the mechanism is unknown, the further reduction in LDL-C levels by omega-3 fatty acid ethyl under statin treatment observed in this study may be of additional benefit in preventing ASCVD.

Just recently, the results of the PROMINENT trial, which prospectively examined the preventive effects of pemafibrate on cardiovascular events among patients with type 2 diabetes, mild-to-moderate hypertriglyceridemia, and low HDL-C and LDL-C levels were published (72). In this trial, pemafibrate was expected to reduce cardiovascular events compared to placebo, however, the study failed to verify it. As the possible cause of the results, the authors discussed the possibility that the increases in apoB and LDL-C levels observed in pemafibrate treated group negated the benefits in the prevention of cardiovascular events that can be obtained from treatment with pemafibrate, such as decreased TG and remnant cholesterol levels. In our PROUD48 study, from baseline to week 16, although we did not observe an increase in apoB levels in PEMA, a significant decrease in apoB levels in OMEGA-3 was observed. Additionally, greater decrease with significant difference in the percentage change from baseline to week 16 in apoB level was observed in OMEGA-3, compared to PEMA. Indeed, in the present study, pemafibrate was shown to be superior to omega-3 fatty acid ethyl in the apoB-48 lowering effect under statin treatment. However, considering the effects of these two agents on apoB and LDL-C levels, we consider that a discussion similar to that of the PROMINENT trial is necessary. Thus, caution is warranted in concluding that pemafibrate is superior to omega-3 fatty acid ethyl in reducing residual ASCVD risk associated with TRLs under statin treatment.

We now demonstrated that pemafibrate is superior to omega-3 fatty acid ethyl in lowering effect of fasting apoB-48. Although we expect that pemafibrate can reduce cardiovascular events involved in the residual ASCVD risk mainly associated with TRLs in patients with dyslipidemia receiving statin treatment compared to omega-3 fatty acid ethyl, there is some concern about the expected superiority of pemafibrate over omega-3 fatty acid ethyl in cardiovascular outcomes associated with residual ASCVD risk as discussed above. On the other hands, in the following points, the results of the PROMINENT trial cannot be directly applied to the perspectives from the PROUD48 study. First, dissimilar to our study, approximately 70% of participants in the PROMINENT trial had a history of cardiovascular disease. Therefore, the PROMINENT trial may be considered as a study that validated the effect of pemafibrate on secondary prevention for ASCVD. Second, as mentioned above, the PROMINENT trial was dominated by participants with previous cardiovascular disease, who had lower baseline LDL-C levels and also lower HDL-C levels than participants in our study. These differences may have attenuated the anti-atherosclerotic effects of pemafibrate on LDL and HDL particle sizes and functions, and may have affected cardiovascular outcomes in the PROMINENT trial. Third, change in fasting TG levels observed in the PROMINENT trial was comparable to our PROUD48 study. However, the effect of pemafibrate on postprandial hypertriglyceridemia or its surrogate marker as a residual ASCVD risk has not been verified in the PROMINENT trial. Therefore, we consider that the results of the PROMINENT trial do not negate the lowering effect of pemafibrate on TRLs-related residual ASCVD risk.

There are several limitations to the present study. First, since we set a surrogate marker fasting apoB-48 that reflects postprandial hypertriglyceridemia, which is one of the residual ASCVD risks, as the primary endpoint, the effects of pemafibrate and omega-3 fatty acid ethyl on atherosclerotic cardiovascular outcomes represented by major adverse cardiovascular events could not be evaluated. Therefore, prospective comparative trial comparing effects of pemafibrate and omega-3 fatty acid ethyl on cardiovascular outcomes are needed. In addition, it is necessary to examine the effects of the two agents on postprandial TRL metabolism in order to compare the detailed mechanisms of their reducing effect on the residual ASCVD risk associated with postprandial hypertriglyceridemia. Second, it had a small sample size and short treatment period. Although we completed a 16-week trial with the required number of participants for this study, further studies with larger sample size and longer treatment period are required. Third, as this was an open-label trial, there were concerns about potential bias and its impact on the results. Finally, the lack of placebo or combination treatment groups is also a limitation in this study.

In conclusion, this is the first randomized trial comparing the lowering effects of pemafibrate and omega-3 fatty acid ethyl on fasting apoB-48. The present study demonstrated that pemafibrate is superior to omega-3 fatty acid ethyl in lowering effect of fasting apoB-48. A prospective comparative trial is needed to evaluate the effects of the two agents on atherosclerotic cardiovascular outcomes.

## Data availability statement

The datasets generated and/or analyzed during the study are available from the corresponding author on reasonable request. Requests to access the datasets should be directed to YT, yktake5@asahikawa-med.ac.jp.

## Ethics statement

The studies involving human participants were reviewed and approved by Certified Review Board of the University of the Ryukyus for Clinical Research Ethics. The patients/participants provided their written informed consent to participate in this study.

## Author contributions

YT, IS, SH, and MO contributed to the participant enrollment and data acquisition. YT had full access to all of the data in this study and takes complete responsibility for the integrity of the data and the accuracy of the data analysis. MS planned and performed the all statistical analyses. YT wrote the first draft of this article. IS, SH, MO, SU, and MS critically revised the manuscript. All authors met the International Committee of Medical Journal Editors criteria for authorship for this manuscript, contributed to developing the study protocol, including the study design, intervention, inclusion and exclusion criteria, variables and endpoints, and have read and approved the final version of this manuscript.
