# Corrigendum: The effects of pemafibrate and Omega-3 fatty acid ethyl on apoB-48 in dyslipidemic patients treated with statin: A prospective, multicenter, open-label, randomized, parallel group trial in Japan (PROUD48 study)

**DOI:** 10.3389/fcvm.2023.1172664

**Published:** 2023-03-16

**Authors:** Yasutaka Takeda, Ichiro Sakuma, Shinya Hiramitsu, Mizuho Okada, Shinichiro Ueda, Masaru Sakurai

**Affiliations:** ^1^ Division of Metabolism and Biosystemic Science, Gastroenterology, and Hematology/Oncology, Department of Medicine, Asahikawa Medical University, Asahikawa, Japan; ^2^ Caress Sapporo Hokko Memorial Clinic, Sapporo, Japan; ^3^ Hiramitsu Heart Clinic, Nagoya, Japan; ^4^ Keiyukai Yoshida Hospital, Asahikawa, Japan; ^5^ Department of Clinical Pharmacology and Therapeutics, University of the Ryukyus, Nishihara, Japan; ^6^ Department of Social and Environmental Medicine, Kanazawa Medical University, Uchinada, Japan

**Keywords:** apolipoprotein B-48, atherosclerotic cardiovascular disease, residual risk, hypertriglyceridemia, pemafibrate, polyunsaturated fatty acids

A Corrigendum on The effects of pemafibrate and Omega-3 fatty acid ethyl on apoB-48 in dyslipidemic patients treated with statin: A prospective, multicenter, open-label, randomized, parallel group trial in Japan (PROUD48 study) By Takeda Y, Sakuma I, Hiramitsu S, Okada M, Ueda S and Sakurai M. (2023) Front. Cardiovasc. Med. 10:1094100. doi: 10.3389/fcvm.2023.1094100

In the published article, there was an error in Table 6 (Adverse events) as published. The error was found in the percentage of participants with sepsis associated with enteritis in the PEMA group. The corrected table appears below.

**Table 6:** Adverse events.

	PEMA (*n* = 63)	OMEGA-3 (*n* = 63)
AEs	9 (14.3)	3 (4.8)
Participants who discontinued treatment because of drug-related AE		
Nausea	1 (1.6)	0 (0.0)
Pain in the extremities	1 (1.6)	0 (0.0)
Erythema on the forearm	1 (1.6)	0 (0.0)
Itching in the forearm	1 (1.6)	0 (0.0)
General malaise	1 (1.6)	0 (0.0)
Anorexia	1 (1.6)	0 (0.0)
Participants who discontinued treatment because of drug-unrelated AE		
Neck stiffness	1 (1.6)	0 (0.0)
Tooth pain	1 (1.6)	0 (0.0)
Sepsis associated with enteritis	1 (1.6)	0 (0.0)
Other AEs		
Worsening of glycemic control	2 (3.2)	1 (1.6)
Acute epiglottitis	1 (1.6)	0 (0.0)
Dental caries	1 (1.6)	0 (0.0)
Liver dysfunction	1 (1.6)	0 (0.0)
Muscular pain	1 (1.6)	0 (0.0)
Fall	0 (0.0)	1 (1.6)
Lumbago	0 (0.0)	1 (1.6)
Elevation of serum CK	0 (0.0)	1 (1.6)

The authors apologize for this error and state that this does not change the scientific conclusions of the article in any way. The original article has been updated.

