# Supplementary material for: The effects of pemafibrate and omega-3 fatty acid ethyl on apoB-48 in dyslipidemic patients treated with statin: A prospective, multicenter, open-label, randomized, parallel group trial in Japan (PROUD48 study)
Source: Front Cardiovasc Med. 2023 Jan 25;10:1094100. doi: 10.3389/fcvm.2023.1094100 (PMC9905248; doi:10.3389/fcvm.2023.1094100)
Supplement: Supplementary file 1 [file Table_1.docx]

Supplementary Table S1: Changes in clinical variables from baseline to week 16 (within-group comparisons).

|  |  | PEMA | | | | |  |  |  | OMEGA-3 | | | | |  |
| --- | --- | --- | --- | --- | --- | --- | --- | --- | --- | --- | --- | --- | --- | --- | --- |
|  | n | Baseline | |  | Week 16 | | *P* |  | n | Baseline | |  | Week 16 | | *P* |
| Body weight (kg) | 58 | 70.4 | 12.3 |  | 70.4 | 12.3 | 0.923 |  | 60 | 69.7 | 15.5 |  | 69.8 | 15.7 | 0.867 |
| Waist circumference (cm) | 57 | 94.0 | 8.8 |  | 93.6 | 8.5 | 0.427 |  | 60 | 92.5 | 10.3 |  | 92.5 | 10.6 | 0.916 |
| BMI (kg/m^2^) | 58 | 26.6 | 3.4 |  | 26.6 | 3.4 | 0.783 |  | 60 | 26.2 | 4.6 |  | 26.3 | 4.7 | 0.468 |
| SBP (mmHg) | 57 | 129.9 | 7.9 |  | 128.9 | 8.2 | 0.405 |  | 60 | 130.0 | 8.4 |  | 129.7 | 8.2 | 0.840 |
| DBP (mmHg) | 57 | 70.8 | 8.0 |  | 73.2 | 14.7 | 0.228 |  | 60 | 68.9 | 8.8 |  | 71.1 | 8.7 | 0.043 |
| Total cholesterol (mg/dL) | 58 | 182.3 | 23.7 |  | 184.4 | 29.4 | 0.550 |  | 61 | 181.6 | 26.9 |  | 169.9 | 28.5 | <0.001 |
| Triglycerides (mg/dL) | 58 | 196 | (76-380) |  | 112 | (59-340) | <0.001 |  | 61 | 218 | (71-492) |  | 153 | (54-475) | <0.001 |
| HDL-C (mg/dL) | 58 | 50.5 | 12.3 |  | 56.7 | 12.6 | <0.001 |  | 61 | 48.9 | 10.8 |  | 51.3 | 13.5 | 0.035 |
| LDL-C (mg/dL) | 58 | 97.6 | 24.3 |  | 109.1 | 28.4 | <0.001 |  | 61 | 96.4 | 22.4 |  | 86.9 | 23.0 | <0.001 |
| Small dense LDL (mg/dL) | 58 | 35.9 | 12.9 |  | 30.0 | 16.5 | 0.001 |  | 61 | 38.6 | 13.8 |  | 32.2 | 11.4 | <0.001 |
| Remnant lipoprotein cholesterol (mg/dL) | 58 | 8.1 | 3.1 |  | 4.1 | 2.6 | <0.001 |  | 61 | 9.2 | 4.4 |  | 6.1 | 4.1 | <0.001 |
| ApoA-I (mg/dL) | 58 | 152.7 | 27.9 |  | 158.4 | 27.2 | 0.059 |  | 61 | 148.8 | 24.0 |  | 146.5 | 24.9 | 0.254 |
| ApoA-II (mg/dL) | 58 | 31.2 | 4.4 |  | 45.3 | 7.4 | <0.001 |  | 61 | 30.6 | 4.9 |  | 28.7 | 4.9 | <0.001 |
| ApoB (mg/dL) | 58 | 89.2 | 17.1 |  | 88.2 | 21.7 | 0.610 |  | 61 | 90.3 | 16.9 |  | 82.7 | 16.8 | <0.001 |
| ApoB-48 (µg/mL) | 58 | 6.7 | (1.5-40.0) |  | 3.3 | (0.8-22.7) | <0.001 |  | 61 | 6.4 | (2.8-40.0) |  | 5.7 | (1.7-36.5) | 0.026 |
| ApoC-II (mg/dL) | 58 | 5.5 | 1.3 |  | 4.4 | 1.5 | <0.001 |  | 61 | 6.3 | 2.0 |  | 5.2 | 1.9 | <0.001 |

| **Supplementary Table S1: Continued.** | | | | | | | | | | | | | | | |
| --- | --- | --- | --- | --- | --- | --- | --- | --- | --- | --- | --- | --- | --- | --- | --- |
|  |  | PEMA | | | | |  |  |  | OMEGA-3 | | | | |  |
|  | n | Baseline | |  | Week 16 | | *P* |  | n | Baseline | |  | Week 16 | | *P* |
| ApoC-III (mg/dL) | 58 | 13.5 | 2.9 |  | 9.1 | 3.0 | <0.001 |  | 61 | 14.8 | 4.2 |  | 13.1 | 4.7 | <0.001 |
| ApoE (mg/dL) | 58 | 4.7 | 1.2 |  | 4.3 | 0.9 | 0.001 |  | 61 | 4.8 | 1.3 |  | 4.4 | 1.3 | 0.001 |
| Fasting plasma glucose (mg/dL) | 58 | 120.9 | 31.6 |  | 114.9 | 27.0 | 0.022 |  | 61 | 116.9 | 21.4 |  | 129.8 | 38.3 | 0.002 |
| HbA1c (%) | 58 | 6.19 | 0.94 |  | 6.27 | 1.07 | 0.323 |  | 61 | 6.16 | 0.82 |  | 6.35 | 1.05 | 0.037 |
| Insulin (IU/L) | 58 | 9.12 | (2.59-110.0) |  | 6.73 | (2.31-59.3) | 0.018 |  | 61 | 9.02 | (1.63-32.0) |  | 9.09 | (0.50-70.0) | 0.438 |
| HOMA-IR | 58 | 2.34 | (0.74-39.4) |  | 1.83 | (0.53-19.5) | 0.007 |  | 61 | 2.70 | (0.39-7.38) |  | 2.80 | (0.38-26.3) | 0.141 |
| HOMA-β (%) | 58 | 60.3 | (9.6-482.9) |  | 62.2 | (11.0-436.3) | 0.518 |  | 61 | 66.3 | (17.3-500.9) |  | 65.7 | (0.74-412.9) | 0.444 |
| AST (IU/L) | 58 | 25 | (13-110) |  | 28 | (13-135) | 0.259 |  | 61 | 25 | (13-97) |  | 25 | (14-76) | 0.112 |
| ALT (IU/L) | 58 | 29 | (13-162) |  | 25 | (8-138) | <0.001 |  | 61 | 25 | (10-174) |  | 31 | (11-146) | 0.127 |
| γ-GTP (IU/L) | 58 | 43 | (11-431) |  | 23 | (9-219) | <0.001 |  | 61 | 36 | (12-545) |  | 43 | (12-666) | 0.673 |
| ALP (IU/L) | 58 | 205 | (24-340) |  | 116 | (22-245) | <0.001 |  | 61 | 227 | (48-462) |  | 204 | (46-318) | <0.001 |
| CK (IU/L) | 58 | 101 | (37-417) |  | 97 | (15-674) | 0.177 |  | 61 | 99 | (42-380) |  | 103 | (35-743) | 0.256 |
| Cr (mg/dL) | 58 | 0.81 | 0.21 |  | 0.84 | 0.20 | 0.013 |  | 61 | 0.83 | 0.20 |  | 0.82 | 0.20 | 0.419 |
| eGFR (mL/min/1.73m^2^) | 58 | 71.4 | 17.1 |  | 68.5 | 15.7 | 0.005 |  | 61 | 67.6 | 15.7 |  | 68.7 | 17.2 | 0.257 |
| Fibrinogen (mg/dL) | 18 | 285.6 | 44.8 |  | 227.3 | 29.4 | <0.001 |  | 15 | 290.9 | 42.3 |  | 288.7 | 59.8 | 0.804 |

**Supplementary Table S1: Changes in clinical variables from baseline to week 16 (within-group comparisons).**

The data are presented as means ± SDs or medians (Min-Max). *P* values are for comparisons between baseline and week 16. BMI: body mass index; SBP: systolic blood pressure; DBP: diastolic blood pressure; HDL-C: high-density lipoprotein cholesterol; LDL-C: low-density lipoprotein cholesterol; Apo: apolipoprotein; HOMA-IR: homeostasis model assessment insulin resistance; HOMA-β: homeostasis model assessment beta-cell function; AST: aspartate aminotransferase; ALT: alanine aminotransferase; γ-GTP: gamma-glutamyl transpeptidase; ALP: alkaline phosphatase; CK: creatine kinase; eGFR: estimated glomerular filtration rate.
