# Supplementary material for: The effects of pemafibrate and omega-3 fatty acid ethyl on apoB-48 in dyslipidemic patients treated with statin: A prospective, multicenter, open-label, randomized, parallel group trial in Japan (PROUD48 study)
Source: Front Cardiovasc Med. 2023 Jan 25;10:1094100. doi: 10.3389/fcvm.2023.1094100 (PMC9905248; doi:10.3389/fcvm.2023.1094100)
Supplement: Supplementary file 2 [file Table_2.docx]

Supplementary Table S2: Changes in clinical variables from baseline to week 16.

|  | PEMA  (n = 58) | | | OMEGA-3  (n = 61) | | | *P* | |
| --- | --- | --- | --- | --- | --- | --- | --- | --- |
|  |  | Mean | SD |  | Mean | SD |  |  |
| Body weight (kg) | - | 0.03 | 2.31 |  | 0.04 | 2.00 |  | 0.855 |
| Waist circumference (cm) | - | 0.41 | 3.82 | - | 0.07 | 4.98 |  | 0.683 |
| BMI (kg/m^2^) | - | 0.01 | 0.92 |  | 0.03 | 0.70 |  | 0.806 |
| SBP (mmHg) | - | 1.07 | 9.64 | - | 0.27 | 10.16 |  | 0.662 |
| DBP (mmHg) |  | 2.50 | 15.30 |  | 2.20 | 8.40 |  | 0.916 |
| Total cholesterol (mg/dL) |  | 2.10 | 26.61 | - | 11.77 | 19.87 |  | 0.002 |
| Triglycerides (mg/dL) | - | 83.34 | 63.53 | - | 62.21 | 73.35 |  | 0.096 |
| HDL-C (mg/dL) |  | 6.21 | 10.07 |  | 2.44 | 8.84 |  | 0.032 |
| LDL-C (mg/dL) |  | 11.50 | 23.58 | - | 9.51 | 16.93 | < | 0.001 |
| Small dense LDL (mg/dL) | - | 5.84 | 13.20 | - | 6.44 | 9.00 |  | 0.774 |
| Remnant lipoprotein cholesterol (mg/dL) | - | 3.98 | 2.99 | - | 3.10 | 4.34 |  | 0.200 |
| ApoA-I (mg/dL) |  | 5.74 | 22.70 | - | 2.25 | 15.22 |  | 0.027 |
| ApoA-II (mg/dL) |  | 14.12 | 6.69 | - | 1.92 | 3.42 | < | 0.001 |
| ApoB (mg/dL) | - | 1.07 | 15.87 | - | 7.57 | 11.69 |  | 0.013 |
| ApoC-II (mg/dL) | - | 1.13 | 1.41 | - | 1.10 | 1.03 |  | 0.893 |
| ApoC-III (mg/dL) | - | 4.39 | 2.55 | - | 1.62 | 2.31 | < | 0.001 |
| ApoE (mg/dL) | - | 0.47 | 0.99 | - | 0.41 | 0.94 |  | 0.746 |
| Fasting plasma glucose (mg/dL) | - | 6.00 | 19.34 |  | 12.82 | 30.28 | < | 0.001 |
| HbA1c (%) |  | 0.07 | 0.57 |  | 0.19 | 0.68 |  | 0.336 |
| Insulin (IU/L) | - | 1.55 | 16.77 |  | 1.98 | 11.17 |  | 0.177 |
| HOMA-IR | - | 0.72 | 5.84 |  | 1.03 | 4.22 |  | 0.062 |
| HOMA-β (%) | - | 1.65 | 93.08 |  | 1.92 | 66.56 |  | 0.810 |
| AST (IU/L) |  | 0.91 | 13.15 |  | 1.07 | 9.26 |  | 0.942 |
| ALT (IU/L) | - | 8.53 | 16.59 |  | 1.66 | 14.34 | < | 0.001 |
| γ-GTP (IU/L) | - | 26.66 | 43.05 |  | 2.38 | 33.11 | < | 0.001 |
| ALP (IU/L) | - | 70.48 | 60.81 | - | 25.80 | 58.03 | < | 0.001 |
| CK (IU/L) |  | 3.00 | 92.12 |  | 12.75 | 92.94 |  | 0.567 |
| Cr (mg/dL) |  | 0.03 | 0.08 | - | 0.01 | 0.08 |  | 0.017 |
| eGFR (mL/min/1.73m^2^) | - | 2.89 | 7.57 |  | 1.13 | 7.69 |  | 0.005 |
| (Fibrinogen) (mg/dL) | - | 58.28 | 47.36 | - | 2.13 | 32.72 |  | 0.001 |

**Supplementary Table S2: Changes in clinical variables from baseline to week 16.**

The data are presented as means ± SDs. *P* values are for comparisons between the two groups. BMI: body mass index; SBP: systolic blood pressure; DBP: diastolic blood pressure; HDL-C: high-density lipoprotein cholesterol; LDL-C: low-density lipoprotein cholesterol; Apo: apolipoprotein; HOMA-IR: homeostasis model assessment insulin resistance; HOMA-β: homeostasis model assessment beta-cell function; AST: aspartate aminotransferase; ALT: alanine aminotransferase; γ-GTP: gamma-glutamyl transpeptidase; ALP: alkaline phosphatase; CK: creatine kinase; eGFR: estimated glomerular filtration rate.
